# Pathways of neighbourhood-level socio-economic determinants of adverse birth outcomes

**DOI:** 10.1186/1476-072X-12-32

**Published:** 2013-06-20

**Authors:** Gang Meng, Mary E Thompson, G Brent Hall

**Affiliations:** 1 School of Planning, University of Waterloo, Waterloo, Ontario, Canada; 2 Department of Statistics and Actuarial Science, University of Waterloo, Waterloo, Ontario N2L3G1, Canada; 3 Esri Canada, 12 Concorde Place, Toronto M3C 3R8, Canada

**Keywords:** Mediation analysis, Pathways of adverse birth outcomes, Neighbourhood-level socio-economic determinants, Preterm birth, Low birth weight

## Abstract

**Background:**

Although socio-economic factors have been identified as one of the most important groups of neighbourhood-level risks affecting birth outcomes, uncertainties still exist concerning the pathways through which they are transferred to individual risk factors. This poses a challenge for setting priorities and developing appropriate community-oriented public health interventions and planning guidelines to reduce the level of adverse birth outcomes.

**Method:**

This study examines potential direct and mediated pathways through which neighbourhood-level socio-economic determinants exert their impacts on adverse birth outcomes. Two hypothesized models, namely the materialist and psycho-social models, and their corresponding pathways are tested using a binary-outcome multilevel mediation analysis. Live birth data, including adverse birth outcomes and person-level exposure variables, were obtained from three public health units in the province of Ontario, Canada. Corresponding neighbourhood-level socio-economic, psycho-social and living condition variables were extracted or constructed from the 2001 Canadian Census and the first three cycles (2001, 2003, and 2005) of the Canadian Community Health Surveys.

**Results:**

Neighbourhood-level socio-economic-related risks are found to have direct effects on low birth weight and preterm birth. In addition, 20-30% of the total effects are contributed by indirect effects mediated through person-level risks. There is evidence of four person-level pathways, namely through individual socio-economic status, psycho-social stress, maternal health, and health behaviours, with all being simultaneously at work. Psycho-social pathways and buffering social capital-related variables are found to have more impact on low birth weight than on preterm birth.

**Conclusion:**

The evidence supports both the materialist and psycho-social conceptualizations and the pathways that describe them, although the magnitude of the former is greater than the latter.

## Background

Birth weight and gestational age are two important indicators of infant health. A growing body of literature contends that low birth weight (LBW) and preterm birth (PTB) are a result of not only direct person-level risks, but also harmful social and environmental exposures during critical stages of foetal development. In addition to environmental influences, socio-economic factors have been consistently identified as some of the most pervasive neighbourhood-level risk factors associated with LBW and PTB incidence [1-4]. Increased use of tobacco, alcohol and illicit drugs, stressful work and living environment, delayed or reduced prenatal care, increased maternal infections, violence and abuse, depression, increased risk of unwanted pregnancy, increased teenage pregnancy, and reduced levels of social and financial support have all been identified as risk factors among low socio-economic status (SES) groups [4].

Despite this knowledge, there is a general lack of understanding of the mechanisms that transfer neighbourhood-level social and environmental conditions to individual instances of adverse birth outcomes. This poses a challenge for setting priorities and developing appropriate public health programs and policies, especially for community health interventions. Two models can be used to disentangle these complex processes.

The psycho-social model [5] argues that relative social position affects individual feelings that, in turn, impact on health status. Extending this argument to birth outcomes implies that different birth outcomes among different socio-economic groups are caused by associated psycho-social stressors [6]. These stressors may cause direct physiological changes in women or in uterine environments and trigger adverse pregnancy outcomes through the interaction of neuroendocrine and immunological processes [7,8]. For example, stress may cause cortisol-induced increases in placental secretion of a corticotrophin-releasing hormone, which increases the production of prostanoids to stimulate uterine contractility and induce preterm birth [9]. Stress activated fight-or-flight response may also disrupt the hypothalamic-pituitaryadrenal axis and thereby trigger the onset of preterm birth [10]. In addition, the release of stress hormones may lead to immunosuppression and alteration of both cellular and humoral immunity, which make the mother susceptible to infection and consequently lead to an adverse birth outcome [11].

Stress may also indirectly increase the risk of LBW or PTB through the development of a depressive self-concept and low commitment to pregnancy [12,13]. In turn, this may lead to careless or unhealthy behaviours during pregnancy, such as smoking [14], heavy alcohol consumption [15], substance abuse [16], delayed or reduced prenatal care [17], and poor dietary intake [18], all of which may increase the risk of LBW and PTB. Since mothers with lower social status tend to have more prolonged exposure to psychological demands and stresses, the psycho-social model may explain the fine “social gradients” in which adverse birth outcomes increase progressively down the social strata [19].

In addition to the psycho-social model, the materialist model [20] argues that birth outcome differences result from different purchasing or controlling powers between social classes. According to this conceptualization, material inequalities, such as income differences, determine the nature of people’s working conditions, living environments, access to health care facilities, and exposure to physical hazards, which may all have an influence on birth outcomes. Research has found that adverse neighbourhood-level living conditions, deprivation, hazardous environments, poor health-related services, and cumulative exposure to income inequality are all significantly associated with adverse birth outcomes [21-25].

The materialist model therefore suggests that birth outcome variations of different social groups are determined by their degree of exposure over the life course to general living environment risks or hazards. In addition, higher-level social-structural factors, such as public service provision, welfare and health coverage, and social and economic policies, may also affect social conditions, living environments and the distribution of resources over social and geographical space. These risks are cumulatively part of the social structure, over which the individual has little control [26]. They may affect a mother’s birth outcomes directly and/or indirectly through the mother’s health behaviour and life style [27].

**Figure 1:**
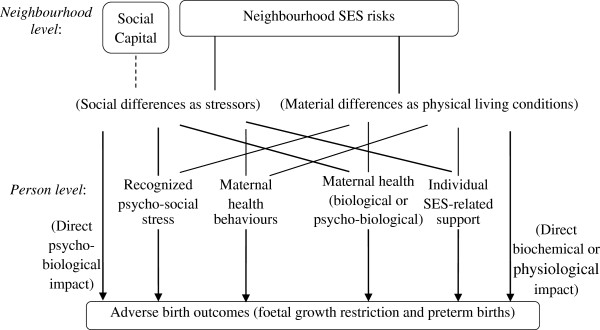
Hypothetical pathways of neighbourhood-level SES risks to adverse birth outcomes.

Based on the psycho-social and materialist models, hypothesized pathways of adverse birth outcomes can be framed as shown in Figure 1. Neighbourhood SES-related differences may both create stressors for residents of lower SES neighbourhoods and result in differences in physical or material conditions. Psycho-social stressors may cause psycho-biological changes and affect directly the uterine environment to induce adverse birth outcomes. Stressors recognized by the mother may lead to maternal psycho-social stresses or depression that result in physiological changes during the pregnancy, or may lead to maternal health behavioural changes that increase the risks of adverse birth outcomes. In addition, if the psycho-social pathways are evident, strong neighbourhood ‘social capital’ [28] may have a buffering effect on adverse birth outcomes, since increased social integration and collective efficacy may help to reduce a mother’s stress and its related negative consequences. The psycho-social and materialist pathways are not mutually exclusive. For example, psycho-social stressors may also cause psycho-biological responses of mothers, which may lead to poor maternal health and consequently adverse birth outcomes. Similarly, material differences such as service unavailability may also lead to behavioural differences or stress.

This paper applies the psycho-social and materialist models to clarify the pathways of psycho-social and material factors on adverse birth outcomes. The pathways actually at work are identified and the extent to which they contribute to foetal growth restriction and preterm birth is evaluated. The hypothesized pathway model shown in Figure 1 is a simplification of reality, which is much more complex than what is shown in the model. For example, given the associations among person-level SES, maternal health, psycho-social stresses, and health behaviours, the person-level factors may affect each other through a very complex process. However, no matter how these factors may enhance the magnitudes or the effects of others, each will end-up with a certain status at the time of pregnancy and may be represented by a factor. Each factor may mediate the influence of neighbourhood-level risks, in a way which represents a distinct and plausible underlying biological mechanism. For example, maternal health conditions may affect oxygen-carrying capacity and uteroplacental blood flow and thus lead to adverse outcomes. Similarly, maternal infections may cause organisms to be transmitted through the placenta and affect fetal growth, and psycho-social stress may result in adverse pregnancy outcomes through the interaction of immunological and neuroendocrine processes. The altered birth outcomes from adverse health behaviours, such as smoking, alcohol use, and substance use, may be due to the exposure of the mother or the fetus to a variety of toxic chemicals, such as nicotine, the metabolite cotinine, carbon monoxide, ethanol, caffeine, and cocaine. Also, lack of SES-related support may lead to lack of nutrient supply to the fetus or exposure to environmental toxins due to poor living conditions.

In the approach and methods discussed next, the separate consideration of each factor in the vertical pathways from neighbourhood-level risks to person-level risks, and hence to adverse birth outcomes, can be identified, and the strength of influence of one pathway to the others can be clarified.

## Methods

### Data collection

To test the psycho-social and materialist model pathways on adverse birth outcomes, outcome data were collected on variables at the individual level, and exposure data were collected for variables at both the individual and neighbourhood levels. In total, data on 90,500 live births (2000–2008), including both singletons and multiple births, were obtained from the Integrated Services for Children Information System (ISCIS) databases of three public health units in Ontario, Canada, namely the Wellington-Dufferin-Guelph (WDG) Health Unit, the Windsor-Essex County (WEC) Health Unit, and the Halton Region Health Unit.

Individual-level variables were collected using the Parkyn postpartum screening tool (adapted from [29]) through the Healthy Babies Healthy Children Program introduced by the Ontario Ministry of Health and Long-term Care (Table 1). Parkyn screening is administered in hospitals and by midwives and is used (with family consent) to screen all newborns for risk. To complement the person-level data, neighbourhood-level SES-related variables were extracted from the 2001 Canadian Census of Population and Housing at the census dissemination area (CDA) level. The relative homogeneity and the sizes of CDAs make these areas suitable to be used as surrogates for neighbourhood units. Other CDA-level SES and psycho-social-related variables that could not be obtained from the Canadian census were constructed from the first three cycles (2001, 2003, and 2005) of the individual-level Canadian Community Health Survey (CCHS) databases [30]. The neighbourhood variables are described in Table 2. The research received ethics clearance from the Office of Research Ethics, University of Waterloo (ORE # 14810).

**Table 1:** Individual-level variable descriptions

***Variable name***	***Description (all variables are Binary:1-yes, 0-no)***
LBW	Low birth weight: weight less than 2,500 grams for a live-born at birth
PTB	Preterm birth: birth before 37 complete weeks of gestation
AGELT19	Maternal age < = 19 at delivery
AGEGT36	Maternal age > =36 at delivery
Female	Baby's sex is female
Multibirth	Multiple births
Lang_not_en	Mother's language is not English
Health_chal	It there any health challenges of the mother
Infections	Infection that can be transmitted in utero and may damage the fetus
Drugs	Drug and alcohol abuse diagnosed in mother
Genetic_chal	Family history of genetic heath challenges that may affect development
Single_parent	Single parent family
No_soc_support	No social support and/or severe isolation related to culture, language and geography
Financ_diffy	Financial difficulties specified by respondent
No_prenatal_care	No prenatal care before six month
Schz_mother	Schizophrenia or bipolar affective disorder of mother
Schz_father	Schizophrenia or bipolar affective disorder of father
Ment_chal_mother	Mental illness/mental challenge in mother
Ment_chal_father	Mental illness/mental challenge in father
Marital_distress	Any marital distress of the mother
LOWEDU	Low education status of the mother specified by respondent
Family_violence	Any family violence specified by respondent
No_preclass	No prenatal class attendance
Stress_delivy	High stress related to delivery
Smoking	Maternal smoking during pregnancy

**Table 2:** CDA-level variable description

***Variable Name***	***Description***
Dwelling_val	Average value of dwellings
Unemploy	Percentage of unemployment
Low_income	Percentage of low income population
Person_perroom	Average persons per room
Person_perbedroom	Average persons per bedroom
Rent_rate	Average rental rate
House_repair	Percentage of houses needing major repair
Low_edu	Percentage of low educated population
Low_ses	Percentage of low socio-economic status (composition of Low_income, unemploy, and low_edu)
Food_insecurity *	Percentage of food insecurity
Insufficent_veg *	Percentage of insufficient vegetable intake
Sense_belong_commu*	Average level of sense of belonging to community (1: strong – 4: weak)
Heavy_drinking *	Percentage of heavy drinkers (0 – 100%)
Emotion_unhappy *	Percentage of emotional unhappiness

* Variables were aggregated to CDAs from interpolated individual CCHS samples.

The two adverse birth outcomes, full-term LBW and PTB, reflect different intra-uterine and perinatal processes and experiences, each of which may have specific implications for foetal development, neonatal morbidity and later childhood functioning. Hence, they are analyzed separately in this paper.

### Data analysis

Three linked steps were used to analyze the proposed pathways of neighbourhood-level determinants of adverse birth outcomes. First, a preliminary multilevel regression analysis was conducted using SAS PROC GLIMMIX to test the respective associations between potential explanatory person- and neighbourhood-level variables and full-term LBW and PTB. PTB was controlled by mother’s age (teenage mother and mother of advanced age), baby’s gender, and multiple births at the person-level to represent age-gender adjusted singleton premature births. LBW was controlled by mother’s age, baby’s gender, and multiple birth, and only full-term live birth data were used for model fitting to represent age-gender adjusted full-term singleton LBW births.

For convenience, only LBW and PTB are used in the following discussion to represent the above adjusted full-term singleton LBW and singleton PTB. In addition to the person-level control variables, a random effect term was included in the models to account for potential neighbourhood-level cluster effects. The person- and neighbourhood-level variables described in Tables 1 and 2 were introduced into the model one at a time to test their respective associations with PTB or LBW. Given the causal hierarchies of adverse birth outcomes and potential correlated within-neighbourhood errors, a multilevel design was used for the analysis since it can effectively model the construction and interaction of aggregations and their members at different levels, while distinguishing their different effects on the dependent variables.

After associated risk variables were identified, a factor analysis was conducted on the person-level risk variables to extract separate factors that represent distinct aspects of person-level risks of LBW and PTB. The results represent a relatively comprehensive set of factors through which neighbourhood-level SES-related risks influence adverse birth outcomes. However, since some of the identified person-level risks represent similar aspects of risk, they are likely to be highly correlated. Using all of these variables in subsequent pathway testing would create potentially unstable models due to multicollinearity and blur the hypothetical pathways shown in Figure 1. Hence, eliminating some of the highly correlated person-level variables from the regression models allows a fit with more stable coefficients and reduces the overall generalizability in terms of the impacts of person-level risks on the dependent variables.

Using the SAS PROC FACTOR procedure, a principal component analysis [31] was first conducted on identified person-level risk variables. The optimal number of factors was determined based on two criteria, namely that each factor should have an eigenvalue greater than the average of the initial communality estimates, and that all of the common variance (defined by the sum of communality estimates) should be explained by the extracted factors. This ensures that the common variance represented by the original personal risk variables can be completely accounted for by the extracted factors without losing any explanatory power. The identified principal factors were then rotated using the varimax orthogonal rotation method to determine the best combination of the person-level risks to represent different aspects of personal characteristics [32]. The orthogonal composite risk factors were then used to test a series of multilevel models of the potential pathways of neighbourhood-level risks on adverse birth outcomes.

Finally, to assess how the total effect (sum of the direct and indirect effects) of exposure to a given neighbourhood-level SES-related variable is transferred through the proposed pathways, mediation analyses for binary-outcome models were conducted following the method described by VanderWeele and Vansteelandt [33]. This method estimates the direct and indirect effects on the odds ratio scale by combining logistic and linear regressions. Several assumptions need to be met for this method to be valid. Specifically, the outcomes need to be rare and the mediator variables continuous. In addition, there should be no unmeasured confounding influence on the total effects, on the mediator-outcome relation, and on the exposure-mediator relation. Since confounding influences on the mediator-outcome relation can be controlled by a comprehensive set of baseline covariates, it is assumed that there is no effect of exposure that confounds the mediator-outcome relation. Also, there should be no interactions between the exposure and mediator variables, and the remaining error in the regression of the mediator and exposure association should be homoscedastic and normally distributed.

PTB rates (about 7% on average) and LBW rates (about 5% on average) describe relatively rare occurrences. The orthogonal person-level factors obtained are continuous and contain all the identified person-level variations in the original data. Hence, it can be reasonably assumed that there are no unmeasured person-level confounders for total effects, for the mediator-outcome relation, and for the exposure-mediator relation. Thus, the majority of the above assumptions are reasonable. The exposure-mediator interaction terms were included in the mediation models to test whether or not there are potential interactions that affect the mediation effects.

The above assumptions are required for a causal interpretation of the fitted models and of the direct and indirect effects. These terms are used here even though the assumptions are not practically verifiable and it is not intended to imply that causality is established in the statistical sense.

The following equations were used to test the mediator-exposure (M-X) association, the exposure-mediator-outcome (X-M-Y) association, and the exposure-outcome (X-Y) association. Since the person-level mediator factors are continuous, the M-X associations were modeled using the Statistical Analysis System (SAS) PROC MIXED procedure as:

$$\mathrm{Facto}r_{1}=\alpha_{0}+\alpha_{1}\mathrm{NBVAR}+e_{\mathrm{ij}}+u_{j}$$

$$\mathrm{Facto}r_{2}=\alpha_{0}+\alpha_{2}\mathrm{NBVAR}+e_{\mathrm{ij}}+u_{j}$$

$$\mathrm{Facto}r_{3}=\alpha_{0}+\alpha_{3}\mathrm{NBVAR}+e_{\mathrm{ij}}+u_{j}$$

(1) $$\mathrm{Facto}r_{4}=\alpha_{0}+\alpha_{4}\mathrm{NBVAR}+e_{\mathrm{ij}}+u_{j},$$

where NBVAR is one of the neighbourhood-level risks identified in the preliminary analysis, *Factor*_*1*_ through *Factor*_*4*_ are person-level risk factors identified in the preceding step and are used to represent the potential person-level pathways shown in Figure 1, *e*_*ij*_ is a person-level random term, and *u*_*j*_ is a neighbourhood-level random term.

The X-M-Y association was modeled using the SAS PROC GLIMMIX procedure as:

$$\mathrm{LBW}\;\left(\mathrm{PTB}\right)\sim \mathrm{binary}\;\left(p_{\mathrm{ij}}\right)$$

Level 1 (personal):

$$\begin{array}{l} \mathrm{logit}\left(p_{\mathrm{ij}}\right)=\beta_{0j}+\beta_{1}\mathrm{AGELT}19_{\mathrm{ij}}+\beta_{2}\mathrm{AGEGT}36_{\mathrm{ij}}\\ \;+\beta_{3}\mathrm{FEMAL}E_{\mathrm{ij}}+\beta_{4}\mathrm{MLTIBIRT}H_{\mathrm{ij}} \end{array}$$

$$+\beta_{6j}\mathrm{Facto}r_{1}+\beta_{7j}\mathrm{Facto}r_{2}+\beta_{8j}\mathrm{Facto}r_{3}+\beta_{9j}\mathrm{Facto}r_{4}$$

Level 2 (neighbourhood):

$$\beta_{0j}=\gamma_{00}+\gamma_{01}\mathrm{NBVAR}+v_{0j}$$

$$\beta_{6j}=\gamma_{10}+\gamma_{11}\mathrm{NBVAR}$$

$$\beta_{7j}=\gamma_{20}+\gamma_{21}\mathrm{NBVAR}$$

$$\beta_{8j}=\gamma_{30}+\gamma_{31}\mathrm{NBVAR}$$

(2) $$\beta_{9j}=\gamma_{40}+\gamma_{41}\mathrm{NBVAR}.$$

In this model, *γ*_*01*_ is treated as the direct effect of *NBVAR* on LBW or PTB. If *γ*_*11*_ through *γ*_*41*_ are 0, so that there are no mediator-exposure interactions, the indirect effect can be obtained as *α*_*1*_*γ*_*10*_ *+ α*_*2*_*γ*_*20*_ *+ α*_*3*_*γ*_*30*_ *+ α*_*4*_*γ*_*40*_ depending on the significance of these parameters, where *α*_*1*_*γ*_*10*_, *α*_*2*_*γ*_*20*_, *α*_*3*_*γ*_*30*_, and *α*_*4*_*γ*_*40*_ represent the respective pathways of SES-related support, psycho-social stress, maternal behaviours, and maternal health, also as described in Figure 1. The potential neighbourhood-level cluster effect was modeled by a neighbourhood-level random term *v*_*0j*_.

The X-Y association was modeled using the SAS PROC GLIMMIX procedure as:

$$\mathrm{LBW}\;\left(\mathrm{PTB}\right)\sim \mathrm{binary}\;\left(p_{\mathrm{ij}}\right)$$

Level 1 (personal):

$$\begin{array}{l} \mathrm{logit}\left(p_{\mathrm{ij}}\right)=\beta_{0j}+\beta_{1}\mathrm{AGELT}19_{\mathrm{ij}}+\beta_{2}\mathrm{AGEGT}36_{\mathrm{ij}}\\ \;+\beta_{3}\mathrm{FEMAL}E_{\mathrm{ij}}+\beta_{4}\mathrm{MLTIBIRT}H_{\mathrm{ij}} \end{array}$$

Level 2 (neighbourhood):

(3) $$\beta_{0j}=c_{00}+c_{01}\mathrm{NBVAR}+v_{0j}.$$

The parameter *c*_*01*_ is the total effect of NBVAR on LBW or PTB. If all the assumptions hold true, the total effect should be approximately equal to the sum of the direct and the indirect effect.

## Results

Based on the first stage of the analysis, statistically significant associations (tested at the 95% confidence level) between person- and neighbourhood-level exposures and the outcomes of PTB and LBW were identified (Table 3). Each of the identified neighbourhood-level risks was used subsequently to test the pathways of their impacts on adverse birth outcomes.

**Table 3:** Regression results for person and neighbourhood-level risks on LBW and PTB

***Level of risk***	***Variables***	***Coeff. for PTB[95% CI]***	***Coeff. for LBW[95% CI]***
Personal risks	Health_chal	1.28[1.06-1.50]	1.14 [0.74-1.53]
	Infections	0.38[0.14-0.63]	-
	Drugs	0.84[0.57-1.11]	1.42 [1.06-1.77]
	Genetic_chal	0.31[0.03-0.59]	**-**
	Single_parent	0.25[0.11-0.39]	0.59 [0.38-0.79]
	No_soc_support	0.32[0.16-0.48]	0.63 [0.40-0.87]
	Financ_diffy	0.37[0.22-0.52]	0.53 [0.31-0.76]
	No_prenatal_care	0.99[0.76-1.21]	0.76 [0.39-1.14]
	Ment_chal_mother	0.59[0.02-1.17]	**-**
	Marital_distress	0.55[0.18-0.92])	0.78 [0.19-1.36]
	Family_violence	0.45[0.01-0.88]	0.97 [0.38-1.56]
	Smoking	0.37[0.22-0.52]	1.10 [0.91-1.29]
	Stress_delivy	1.56[1.47-1.66]	0.87 [0.69-1.05]
	Lowedu	**-**	0.64 [0.38-0.91]
Neighbourhood risks	Low_income	0.71[0.42-1.00]	1.09[0.67-1.51]
	Low_edu	0.35[0.18-0.52]	0.58[0.31-0.84]
	Unemploy	0.01[0.01-0.02]	0.02[0.01-0.03]
	Low_ses	1.00[0.64-1.36]	1.60[1.06-2.14]
	DWL_VAL	−0.13[−0.17--0.09]	−0.16[−0.22--0.09]
	Rent_rate	0.31[0.18-0.45]	0.65[0.45-0.84]
	House_repair	1.12[0.52-1.71]	**-**
	Food_insecurity	1.31[0.78-1.83]	2.19[1.40-2.98]
	Insufficent_veg	0.89[0.44-1.34]	1.57[0.87-2.28]
	Heavy_drinking	−0.59[−1.11--0.07]	−0.93[−1.77--0.09]
	Person_perroom	**-**	−0.41[−0.59--0.23]
	Person_ perbedroom	**-**	−1.34[−1.82--0.86]
	Emotion_unhappy	**-**	3.57[0.82-6.31]

Note: Coefficient values in this table are the original regression coefficients in log scale calculated by the multilevel models described in the preliminary analysis section. See the method in this section for detailed description on outcome variables and controlling variables.

Many of the identified person-level risk variables for maternal health, behaviour, social and financial support, psycho-social, and genetic aspects shown in Table 3 are the same for LBW and PTB, with some minor variations. To identify these unique aspects and to control for multicollinearity, orthogonal risk factors were constructed separately for LBW and PTB based on their corresponding identified person-level risk variables.

The factor patterns are listed in Tables 4 and 5, where only large factor loadings (> = 0.3) are displayed so that the major contribution of risk variables to each factor can be clearly shown. Although factor loadings less than 0.3 are not displayed in the tables, they were still used to estimate the factor scores even though their impacts on corresponding factors were small. As suggested in the previous section, the factors which form the explanatory variables for the pathway models are continuous and centred with 0 means and are approximately orthogonal.

**Table 4:** Factor pattern for person-level variables associated with PTB

***Factors***	***Factor***_***1 ***_***(SES-related support)***	***Factor***_***2 ***_***(Psycho-social stress)***	***Factor***_***3 ***_***(Behavioural)***	***Factor***_***4 ***_***(Maternal health)***	***Factor***_***5 ***_***(Genetic)***
***Variables***					
Health_chal	.	.	.	0.50	.
Infections	.	.	0.42	.	.
Drugs	0.38	.	0.61	.	.
Genetic_chal	.	.	.	.	0.68
Single_parent	0.62	0.41	0.38	.	.
No_soc_support	0.77	.	.	.	.
Financ_diffy	0.72	.	0.33	.	.
No_prenatal_care	0.51	.	0.41	.	.
Ment_chal_mother	0.32	.	.	.	0.62
Marital_distress	.	0.79	.	.	.
Family_violence	.	0.67	0.35	.	.
Stress_delivy	.	.	.	0.48	.
Smoking	.	0.34	0.51	.	.

**Table 5:** Factor pattern for person-level variables associated with LBW

***Factors***	***Factor***_***1 ***_***(SES-related support)***	***Factor***_***2 ***_***(Psycho-social stress)***	***Factor***_***3 ***_***(Behavioural)***	***Factor***_***4 ***_***(Maternal health)***
***Variables***				
Health_chal	.	.	.	0.54
Drugs	0.40	.	0.53	.
Single_parent	0.59	0.39	0.43	.
No_soc_support	0.75	.	.	.
Financ_diffy	0.68	.	0.45	.
No_prenatal_care	0.52	.	0.43	.
Marital_distress	.	0.77	.	.
Lowedu	.	0.31	0.60	.
Family_violence	.	0.62	0.44	.
Stress_delivy	.	.	.	0.46
Smoking	.	.	0.74	.

As shown in Table 4, *Factor*_*1*_ is mainly composed of single parent, no social support, financial difficulty, and no prenatal care, all of which represent a lack of socio-economic resources. Thus, *Factor*_*1*_ is interpreted as a SES-related support factor. *Factor*_*2*_ is mainly composed of single parent, marital distress, family violence, and smoking. These variables have a common characteristic of representing high stress or depression of the mother resulting from recognized family stressors. Since the data do not contain directly measured maternal depression, there might be other potential unmeasured or unconscious psycho-social stresses caused by chronic living or working stressors. *Factor*_*2*_ is therefore a recognized family psycho-social stress factor. *Factor*_*3*_ is mainly composed of drug use, no prenatal care, infections, and smoking, and somewhat related to single parent, family violence, and financial difficulties. The common characteristic among these variables is that they relate to risky behaviours of the mother. Hence, this factor is a behavioural factor. *Factor*_*4*_ is mainly composed of mother’s health challenges and stress related to delivery. Hence, this represents a maternal health factor. *Factor*_*5*_ is composed of family history of genetic health challenges, and mentally challenged mother (which may also be genetically inherited), and is considered to be a genetic factor.

Similarly, for LBW (Table 5), *Factor*_*1*_ is an SES-related support factor since all the components, including no social support, financial difficulties, single parenthood, no prenatal care and drug use, are associated with low SES. *Factor*_*2*_ is a recognized family psycho-social stress factor since the major components are marital distress, family violence, stress related to delivery and low education. *Factor*_*3*_ is mainly composed of maternal drug use, smoking, low education, single parenthood, financial difficulty, no prenatal care and family violence, and is considered to be a behavioural factor. *Factor*_*4*_ is a maternal health factor since it is composed of maternal health challenges and stress related to delivery.

It is evident in Tables 4 and 5 that one variable can contribute to several factors. For example, the SINGLE_PARENT contributes to SES-related support, recognized family psycho-social stress and health behaviour. This adds support for the point made earlier that person-level determinants are likely to be interrelated. A single mother may have low SES status and increased psycho-social stress leading to adverse health behaviours. Hence, while the current variables may measure several different aspects of person-level risks, the identified factors allow these different aspects to be separated from each other to represent the current status of these aspects after the pathways among person-level risks have taken effect.

A multilevel regression analysis using the method described earlier was conducted to test the associations between PTB and LBW and their respective factors. The results show that PTB is associated with *Factor*_*1*_ through *Factor*_*4*_. However, *Factor*_*5*_ is not statistically associated with PTB at the 5% significance level, suggesting a lack of association between PTB and family genetic history. This suggests that PTB may be caused not so much by direct genetic differences as by the consequences or difficulties brought about by prior genetic health problems. LBW is significantly associated with all four of its factors. Hence, both dependent variables are associated with four factors, which have different loadings in the two cases.

Based on these results, the first four factors for PTB and all four factors for LBW were used in the mediation analysis. These factors represent unique aspects of person-level determinants of adverse birth outcomes, including person-level SES-related support, recognized psycho-social stresses, health behaviours, and mother’s health. Being orthogonal and continuous, they overcome the issue of multicollinearity without losing generalizability.

Mediation analysis was conducted for each of the identified neighbourhood risks (Table 3). Results estimated from Equations 1, 2, and 3, including the significant individual indirect effects (*α*_*1*_*γ*_*10*_, *α*_*2*_*γ*_*20*_, *α*_*3*_*γ*_*30*_, and *α*_*4*_*γ*_*40*_), the sum of indirect effects, the direct effect (*γ*_*01*_ in Equation 2), the total effect (*c*_*01*_ in Equation 3), and the moderation effects (γ_11_, γ_21_, γ_31_, and γ_41_ in Equation 2, if present) for each neighborhood-level variable, are listed in Tables 6 and 7 for PTB and LBW respectively. The random terms *u*_*j*_ and *e*_*ij*_ in Equation 1 were also calculated. In general, the values of *u*_*j*_ are about 1% of *e*_*ij*_ and the standard errors for the *e*_*ij*_ variances are very small. The assumption that the errors for the M-X association models (Equation 1) are normally distributed (with constant variance) is approximately satisfied for the direct and indirect effect estimations.

**Table 6:** Mediation analysis results for PTB

***Neighbourhood variables***	***Pathways***	***Coefficient***	***Effect of individual pathway [95% ci]***	***Effect [95% ci]***
**Low_ses**	Indirect	Through SES-related support: *α*_*1*_*γ*_*10*_	0.115 [0.071-0.158]	0.253[0.197-0.308]
		Through psycho-social: *α*_*2*_*γ*_*20*_	0.021 [0.011-0.031]	
		Through behavioural: *α*_*3*_*γ*_*30*_	0.054 [0.036-0.072]	
		Through health: *α*_*4*_*γ*_*40*_	0.063 [0.035-0.091]	
	Direct	*γ*_*01*_		0.641[0.281-1.001]
	Indirect + direct			0.8935
	Total	*c*_*01*_		0.981[0.626-1.337]
**Low_edu**	Indirect	Through SES-related support: *α*_*1*_*γ*_*10*_	0.024 [0.012-0.036]	0.051[0.036-0.067]
		Through psycho-social: *α*_*2*_*γ*_*20*_	0.008 [0.004-0.012]	
		Through behavioural: *α*_*3*_*γ*_*30*_	0.019 [0.011-0.028]	
	Direct	*γ*_*01*_		0.305[0.136-0.475]
	Indirect + direct			0.356
	Total	*c*_*01*_		0.351[0.182-0.521]
**Low_income**	Indirect	Through SES-related support: *α*_*1*_*γ*_*10*_	0.110 [0.069-0.152]	0.213[0.164-0.262]
		Through psycho-social: *α*_*2*_*γ*_*20*_	0.013 [0.006-0.019]	
		Through behavioural: *α*_*3*_*γ*_*30*_	0.023 [0.012-0.034]	
		Through health: *α*_*4*_*γ*_*40*_	0.068 [0.044-0.091]	
	Direct	*γ*_*01*_		0.425[0.132-0.718]
	Indirect + direct			0.638
	Total	*c*_*01*_		0.705[0.418-0.992]
**Unemploy**	Indirect	Through SES-related support: *α*_*1*_*γ*_*10*_	0.002 [0.001-0.003]	0.004[0.003-0.005]
		Through psycho-social: *α*_*2*_*γ*_*20*_	0.0002 [0.0-0.0004]	
		Through behavioural: *α*_*3*_*γ*_*30*_	0.001 [0.00-0.001]	
		Through health: *α*_*4*_*γ*_*40*_	0.002 [0.001-0.002]	
	Direct	*γ*_*01*_		0.007[0.000-0.015]
	Indirect + direct			0.012
	Total	*c*_*01*_		0.013[0.005-0.02]
**Rent_rate**	Indirect	Through SES-related support: *α*_*1*_*γ*_*10*_	0.050 [0.032-0.068]	0.089[0.067-0.112]
		Through psycho-social: *α*_*2*_*γ*_*20*_	0.005 [0.003-0.008]	
		Through behavioural: *α*_*3*_*γ*_*30*_	0.013 [0.008-0.018]	
		Through health: *α*_*4*_*γ*_*40*_	0.021 [0.009-0.033]	
	Direct	*γ*_*01*_		0.228[0.092-0.365]
	Indirect + direct			0.318
	Total	*c*_*01*_		0.311[0.177-0.445]
	Moderation: Health *RENT_RATE	*γ*_*41*_		−0.183[−0.311--0.055]
**House_repair**	Indirect	Through SES-related support: *α*_*1*_*γ*_*10*_	0.073 [0.036-0.110]	0.118[0.066-0.17]
		Through psycho-social: *α*_*2*_*γ*_*20*_	0.013 [0.003-0.024]	
		Through behavioural: *α*_*3*_*γ*_*30*_	0.031 [0.00-0.066]	
	Direct	*γ*_*01*_		0.912[0.313-1.51]
	Indirect + direct			1.03
	Total	*c*_*01*_		1.117[0.522-1.712]
	Moderation: Behavioral *HOUSE_REPAIR	*γ*_*31*_		0.549[−0.023-1.12]
**Dwelling _val**	Indirect	Through SES-related support: *α*_*1*_*γ*_*10*_	−0.007 [−0.01--0.004]	−0.02[−0.025--0.016]
		Through psycho-social: *α*_*2*_*γ*_*20*_	−0.002 [−0.003--0.001]	
		Through behavioural: *α*_*3*_*γ*_*30*_	−0.004 [−0.005--0.002]	
		Through health: *α*_*4*_*γ*_*40*_	−0.008 [−0.011--0.005]	
	Direct	*γ*_*01*_		−0.093[−0.132--0.053]
	Indirect + direct			−0.1127
	Total	*c*_*01*_		−0.126[−0.166--0.085]
**Food_insecurity**	Indirect	Through psycho-social: *α*_*2*_*γ*_*20*_	0.018 [0.007-0.028]	0.153[0.106-0.199]
		Through behavioural: *α*_*3*_*γ*_*30*_	0.049 [0.029-0.068]	
		Through health: *α*_*4*_*γ*_*40*_	0.087 [0.045-0.128]	
	Direct	*γ*_*01*_		0.907[0.382-1.433]
	Indirect + direct			1.06
	Total	*c*_*01*_		1.301[0.779-1.823]
**Insufficent_veg**		Through SES-related support: *α*_*1*_*γ*_*10*_	0.062 [0.036-0.087]	0.194[0.148-0.239]
		Through psycho-social: *α*_*2*_*γ*_*20*_	0.008 [0.001-0.016]	
		Through behavioural: *α*_*3*_*γ*_*30*_	0.024 [0.01-0.038]	
		Through health: *α*_*4*_*γ*_*40*_	0.10 [0.066-0.134]	
	Direct	*γ*_*01*_		0.634[0.19-1.078]
	Indirect + direct			0.828
	Total	*c*_*01*_		0.88[0.435-1.325]
**Heavy_drinking**	Indirect	Through SES-related support: *α*_*1*_*γ*_*10*_	−0.048 [−0.071--0.024]	−0.135[−0.184--0.086]
		Through psycho-social: *α*_*2*_*γ*_*20*_	−0.010 [−0.019--0.002]	
		Through behavioural: *α*_*3*_*γ*_*30*_	−0.024 [−0.04--0.007]	
		Through health: *α*_*4*_*γ*_*40*_	−0.053 [−0.093--0.014]	
	Direct	*γ*_*01*_		−0.421[−0.937-0.094]
	Indirect + direct			−0.556
	Total	*c*_*01*_		−0.59[−1.111--0.07]

**Note**: In this and Table 7, α1*, α*_*2*_*, α*_*3*_*,*and *α*_*4*_ are the regression coefficients of a neighbourhood-level exposure to the respective person-level factor outcomes calculated in Equation 1. *γ*_*10*_*, γ*_*20*_*, γ*_*30*_*,*and *γ*_*40*_ are the fixed main effects (original regression coefficients in log scale) of corresponding person-level factors to an adverse birth outcome calculated in Equation 2. *α*_*1*_*γ*_*10*_*, α*_*2*_*γ*_*20*_*, α*_*3*_*γ*_*30*_*,* and *α*_*4*_*γ*_*40*_ are the products of the above coefficients, representing the indirect effects through SES-related support, psycho-social, behavioural and health pathways respectively. *γ*_*01*_ represents the direct effect of a neighbourhood-level exposure to the adverse birth outcome (original regression coefficients in log scale calculated in Equation 2). *c*_*01*_ represents the total effect of a neighbourhood-level exposure to the adverse birth outcome (original regression coefficients in log scale calculated in Equation 3). See Methods for the three equations in the mediation analysis.

**Table 7:** Mediation analysis results for LBW

***Neighbourhood variables***	***Pathways***	***Coefficient***	***Effect of individual pathway [95% ci]***	***Effect [95% ci]***
**Dwelling_val**	Indirect	Through SES-related support: *α*_*1*_*γ*_*10*_	−0.006 [−0.009--0.004]	−0.026 [−0.031--0.021]
		Through psycho-social: *α*_*2*_*γ*_*20*_	−0.005 [−0.007--0.003]	
		Through behavioural: *α*_*3*_*γ*_*30*_	−0.012 [−0.016--0.008]	
		Through health: *α*_*4*_*γ*_*40*_	−0.002 [−0.004--0.001]	
	Direct	*γ*_*01*_		−0.123 [−0.19--0.056]
	Indirect + direct			−0.149
	Total	*c*_*01*_		−0.156 [−0.224--0.088]
	Moderation: Behavioural * Dwelling_val	*γ*_*31*_		0.0578 [0.001-0.115]
**Person_perroom**	Indirect	Through SES-related support: *α*_*1*_*γ*_*10*_	−0.013 [−0.024--0.003	−0.031 [−0.044--0.018]
		Through psycho-social: *α*_*2*_*γ*_*20*_	−0.004 [−0.007--0.001]	
		Through behavioural: *α*_*3*_*γ*_*30*_	−0.013 [−0.02--0.007]	
	Direct	*γ*_*01*_		−0.338 [−0.517--0.158]
	Indirect + direct			−0.368
	Total	*c*_*01*_		−0.406 [−0.587--0.225]
**Person_perbedroom**	Indirect	Through SES-related support: *α*_*1*_*γ*_*10*_	−0.047 [−0.077--0.017]	−0.133 [−0.172--0.095]
		Through psycho-social: *α*_*2*_*γ*_*20*_	−0.02 [−0.029--0.01]	
		Through behavioural: *α*_*3*_*γ*_*30*_	−0.049 [−0.067--0.03]	
		Through health: *α*_*4*_*γ*_*40*_	−0.018 [−0.031--0.005]	
	Direct	*γ*_*01*_		−1.095 [−1.569--0.621]
	Indirect + direct			−1.228
	Total	*c*_*01*_		−1.34 [−1.816--0.863]
**Rent_rate**	Indirect	Through SES-related support: *α*_*1*_*γ*_*10*_	0.034 [0.0118-0.056]	0.082 [0.056-0.108]
		Through psycho-social: *α*_*2*_*γ*_*20*_	0.012 [0.007-0.017]	
		Through behavioural: *α*_*3*_*γ*_*30*_	0.029 [0.019-0.038]	
		Through health: *α*_*4*_*γ*_*40*_	0.007 [0.00-0.014]	
	Direct	*γ*_*01*_		0.549 [0.347-0.751]
	Indirect + direct			0.631
	Total	*c*_*01*_		0.645 [0.447-0.843]
	Moderation: Health *RENT_RATE	*γ*_*41*_		−0.355 [−0.554--0.157]
**Low_income**	Indirect	Through SES-related support: *α*_*1*_*γ*_*10*_	0.08 [0.028-0.131]	0.202 [0.143-0.26]
		Through psycho-social: *α*_*2*_*γ*_*20*_	0.029 [0.018-0.04]	
		Through behavioural: *α*_*3*_*γ*_*30*_	0.058 [0.038-0.077]	
		Through health: *α*_*4*_*γ*_*40*_	0.035 [0.019-0.052]	
	Direct	*γ*_*01*_		0.861 [0.431-1.292]
	Indirect + direct			1.063
	Total	*c*_*01*_		1.09 [0.667-1.512]
	Moderation: Health *LOW_INCOME	*γ*_*41*_		−0.645[−1.045--0.253]
**Unemploy**	Indirect	Through SES-related support: *α*_*1*_*γ*_*10*_	0.001 [0.0-0.002]	0.004 [0.003-0.005]
		Through psycho-social: *α*_*2*_*γ*_*20*_	0.001 [0.00-0.001]	
		Through behavioural: *α*_*3*_*γ*_*30*_	0.001 [0.001-0.002]	
		Through health: *α*_*4*_*γ*_*40*_	0.001 [0.00-0.001]	
	Direct	*γ*_*01*_		0.015 [0.004-0.026]
	Indirect + direct			0.019
	Total	*c*_*01*_		0.02 [0.009-0.031]
	Moderation: Health * UNEMPLOY	*γ*_*41*_		−0.014 [−0.024--0.004]
**Low_edu**	Indirect	Through SES-related support: *α*_*1*_*γ*_*10*_	0.012 [0.002-0.021]	0.135 [0.084-0.187]
		Through psycho-social: *α*_*2*_*γ*_*20*_	0.02 [0.013-0.027]	
		Through behavioural: *α*_*3*_*γ*_*30*_	0.104 [0.054-0.154]	
	Direct	*γ*_*01*_		0.437 [0.171-0.702]
	Indirect + direct			0.572
	Total	*c*_*01*_		0.577 [0.31-0.843]
**Low_ses**	Indirect	Through SES-related support: *α*_*1*_*γ*_*10*_	0.073 [0.026-0.119]	0.265 [0.203-0.328]
		Through psycho-social: *α*_*2*_*γ*_*20*_	0.051 [0.035-0.068]	
		Through behavioural: *α*_*3*_*γ*_*30*_	0.124 [0.088-0.159]	
		Through health: *α*_*4*_*γ*_*40*_	0.018 [0.002-0.033]	
	Direct	*γ*_*01*_		1.177 [0.629-1.725]
	Indirect + direct			1.442
	Total	*c*_*01*_		1.601 [1.059-2.143]
**Emotion_unhappy**	Indirect	Through SES-related support: *α*_*1*_*γ*_*10*_	0.143 [0.047-0.239]	0.394 [0.253-0.536]
		Through psycho-social: *α*_*2*_*γ*_*20*_	0.077 [0.026-0.128]	
		Through behavioural: *α*_*3*_*γ*_*30*_	0.175 [0.083-0.266]	
	Direct	*γ*_*01*_		2.873 [0.156-5.591]
	Indirect + direct			3.268
	Total	*c*_*01*_		3.566 [0.823-6.309]
**Food_insecurity**	Indirect	Through SES-related support: *α*_*1*_*γ*_*10*_	0.097 [0.036-0.159]	0.314 [0.235-0.392]
		Through psycho-social: *α*_*2*_*γ*_*20*_	0.045 [0.027-0.063]	
		Through behavioural: *α*_*3*_*γ*_*30*_	0.124 [0.085-0.163]	
		Through health: *α*_*4*_*γ*_*40*_	0.047 [0.024-0.071]	
	Direct	*γ*_*01*_		1.801 [1.007-2.596]
	Indirect + direct			2.115
	Total	*c*_*01*_		2.186 [1.396-2.977]
	Moderation: Health * FOOD_INSECURITY	*γ*_*41*_		−1.073 [−1.82--0.326]
**Insufficent_veg**	Indirect	Through SES-related support: *α*_*1*_*γ*_*10*_	0.039 [0.013-0.065]	0.172 [0.13-0.215]
		Through psycho-social: *α*_*2*_*γ*_*20*_	0.022 [0.009-0.035]	
		Through behavioural: *α*_*3*_*γ*_*30*_	0.05 [0.027-0.074]	
		Through health: *α*_*4*_*γ*_*40*_	0.061 [0.04-0.082]	
	Direct	*γ*_*01*_		1.344 [0.644-2.044]
	Indirect + direct			1.516
	Total	*c*_*01*_		1.574 [0.865-2.282]
**Heavy_drinking**	Indirect	Through SES-related support: *α*_*1*_*γ*_*10*_	−0.025 [−0.045--0.006]	−0.134 [−0.176--0.092]
		Through psycho-social: *α*_*2*_*γ*_*20*_	−0.025 [−0.04--0.01]	
		Through behavioural: *α*_*3*_*γ*_*30*_	−0.053 [−0.08--0.027]	
		Through health: *α*_*4*_*γ*_*40*_	−0.031 [−0.052--0.009]	
	Direct	*γ*_*01*_		−0.748 [−1.575-0.08]
	Indirect + direct			−0.882
	Total	*c*_*01*_		−0.927 [−1.768--0.085]

As shown in Tables 6 and 7, there is no mediator-exposure interaction (moderation effect) for most of the variables examined, with a few exceptions (behaviour interacts with houses in need of repair and maternal health interacts with rent rate for PTB; behaviour interacts with dwelling value and maternal health interacts with rent rate, low income, unemployment, and food insecurity for LBW). For the variables with interaction effects, although their estimated indirect effects for corresponding pathways may be somewhat biased, the biases for the sum of the indirect effects are likely to be very small given that only one out of several pathways an the interaction effect present.

## Discussion

The statistically significant direct effects (shown by 95% confidence intervals in Tables 6 and 7) indicate that all neighbourhood-level SES-related risks remained singly associated with PTB and LBW after controlling for person-level risk factors, except for the case of neighbourhood-level heavy drinking and PTB. It can be seen, by comparing the direct effect with the sum of the indirect effects for each variable, that the former are much greater than the latter for almost all neighbourhood-level variables. Taking into account potential unmeasured person-level determinants and measurement errors, these larger values may still suggest a major pathway of neighbourhood-level SES-related variables to LBW and PTB through direct biological or psycho-biological impacts on pregnancy. This finding provides support for the direct pathways described in Figure 1.

Although less dramatic in terms of the fitted models, the neighbourhood-level SES-related risks also exert impacts indirectly on LBW and PTB through the mother’s person-level risk factors. It is evident in Tables 6 and 7 that indirect effects account for approximately 20-30% of the total effects for these variables. Directly measured neighbourhood-level SES variables, including low income, unemployment, education, low SES and dwelling value, affect the mother’s person-level risks through all four pathways, namely SES-related support, recognized psycho-social stress, health behaviours, and maternal health. The only difference at the neighbourhood level is that a higher proportion of lower educated residents does not directly lead to a higher proportion of health-challenged mothers and consequent LBW or PTB. Rather, low educational environment appears to exert an impact on LBW and PTB mainly through the other three personal pathways.

Comparing each individual indirect effect with the sum of the indirect effects shown in Table 6, an obvious major indirect pathway for PTB is the person-level SES-related support pathway, which accounts for approximately 50% of the overall indirect effects. The next most influential pathway is maternal health, which is the major pathway for neighbourhood-level dwelling values, food insecurity, and insufficient vegetable intake, and the second major pathway for low neighbourhood SES, income, and unemployment and rent rates. The behaviour pathway is the third most influential, and the psycho-social pathway has the least influential effect for PTB. These results indicate that the materialist pathways may have a relatively more important impact on PTB than the psycho-social pathways.

The pathways show a relatively more complex pattern for LBW than PTB. It can also be seen in Table 7 that the behavioural pathway and the SES-related support pathway are equally influential in their impacts on LBW. Although less dramatic than these two pathways, the psycho-social pathway shows a much greater impact on LBW than on PTB. This pathway has the second most influential effect for low education rates, and the third most influential pathway for average neighbourhood dwelling values, average neighbourhood-level person per room, person per bedroom, rent rate, low SES, and emotional unhappiness. Thus, given the important roles played by the behavioural and psycho-social pathways, the psycho-social impacts appear to exert a more important effect on LBW than on PTB, although the materialist impact is still prevalent.

For neighbourhood living conditions, the maternal health pathways play less dramatic roles than expected in terms of their impacts on LBW and PTB. The impacts of average person per room on LBW and houses needing major repairs on PTB are not mediated by maternal health, and the impacts of average person per bedroom, rent rate, and dwelling values on LBW are barely mediated by maternal health. In addition, average rent rate shows a negative moderation effect on the impact of maternal health on LBW and PTB, and neighbourhood-level low income, unemployment, food insecurity show negative moderation effects on the impacts of maternal health on LBW.

The negative impact of a poor living environment on personal health or chronic conditions may take years to develop and accumulate. Hence, the consequences of a poor living environment may not yet have emerged for mothers-to-be given their relatively young ages. The other plausible explanation is that the negative impacts of poor living conditions may be partially absorbed by the mother and less likely to pass on to the foetus. Thus, the SES-related support, behavioural and psycho-social pathways may play more important roles than the maternal health pathway for the impacts of living conditions on adverse birth outcomes.

These neighbourhood-level effects are manifested in several ways. For example, neighbourhoods with a high proportion of houses in need of repair are likely to represent relatively old and deteriorated areas. Hence, they may be demographically unstable, “invisible” to outsiders, and likely to have a reputation for social disorder and crime. All of these factors may increase localized stresses and be associated with unhealthy behaviours of local residents. Thus, it is not surprising to observe in Table 6 that, in addition to its direct environmental impact on PTB, the influence of houses in need of repair is mediated by mothers’ SES, health behaviours, and psycho-social risks. It also has a positive moderation effect on the impact of maternal behaviours on PTB. This means that adverse health behaviours during pregnancy are associated with a more elevated risk of PTB for women living in deteriorated neighbourhoods than for those living in better quality areas.

High rates of food insecurity and insufficient vegetable intake are also closely associated with low neighbourhood-level SES. It can be argued that insufficient vegetable intake may be due to poor personal diet choices, and food insecurity is simply the result of a lack of personal purchasing power. However, personal food choices and purchasing behaviours can also be determined by food availability. It can be seen from Tables 6 and 7 that the major pathway of these two variables is through maternal health, followed by health behaviours, indicating that food insecurity or lack of a healthy food supply may affect adverse birth outcomes directly through maternal health and indirectly through influencing mothers’ choice of a less healthy diet. Hence, compared to the above living condition variables, neighbourhood-level supplies of healthy food may have a more immediate impact on maternal health than living conditions on the consequential adverse birth outcomes.

As discussed earlier, the psycho-social pathways are the least influential pathways in terms of magnitude, especially for PTB. However, although its coefficients are small, the psycho-social pathway does mediate almost all neighbourhood-level impacts on PTB and LBW. Other pathways, such as the behavioural pathways and direct effects, may also potentially be affected by psycho-social stressors or psycho-biological changes. These strands of evidence support the psycho-social pathway model and suggest that strong neighbourhood social capital is likely to have a buffering effect on adverse birth outcomes.

For PTB, direct measures of social capital, such as the average level of sense of belonging to a local community and emotional unhappiness, do not show an association with PTB, although a sense of belonging is only marginally insignificant at the 5% level (−1.78, p = 0.14). Neighbourhoods with heavy drinking rates may represent more social activities. Hence, it is possible that these neighbourhoods are actually better socially integrated, more internally coherent for residents, and with stronger social ties than low drinking rate neighbourhoods. Perhaps counter-intuitively, Table 6 indicates that mothers in high heavy drinking rate neighbourhoods are healthier, and have higher SES, better psycho-social status, and generally healthier behaviours. This result may indirectly support the existence of a buffering effect of such neighbourhoods on the impact of psycho-social stresses on PTB.

Average level of emotional unhappiness exhibits a tendency towards buffering LBW outcomes. Higher neighbourhood-level emotional unhappiness is associated with higher LBW after controlling for personal risks. It also indirectly affects LBW through increased personal psycho-social stress, adverse health behaviours and low SES. As discussed earlier, higher neighbourhood heavy drinking rates may represent increased neighbourhood social activities and increased social ties. Hence, this factor also apparently helps to reduce the risks of having a LBW child.

It should be noted that a neighbourhood with a high heavy drinking rate does not necessarily mean that the mothers in this neighbourhood are heavy drinkers. While a mother’s heavy drinking is likely to be harmful for the mother’s pregnancy outcomes, non-heavy-drinking mothers in such a neighbourhood benefit from living in a more integrated social environment. The results in Table 3 do show that drug and alcohol use are positively associated with both PTB and LBW. This underlines the importance of distinguishing neighbourhood-level and person-level determinants for drawing appropriate inferences.

## Conclusions

The results of this paper are specific to the data set used, but the methodology and approach are applicable in other countries and other contexts, given availability of the same kind of data. It is highly likely that the models which emerge would be different in some details in other geographic areas. Nevertheless, there are likely to be commonalities among the areas consistent with the predictions of theory for the impacts of risk factors associated with SES.

Based on the above discussion of results, it can be concluded that neighbourhood-level SES-related risks exert their impacts on adverse birth outcomes through all four hypothesized person-level pathways and the direct effect, although neighbourhood-level psycho-social factors seem to play a more important role in LBW outcomes than in the case of PTB. Hence, both the psycho-social and materialist models mentioned earlier, as complementary explanations, are supported.

However, before drawing any overarching conclusions and suggestions from the results presented in the previous section, it is important to reiterate that the cross-sectional and observational nature of this study limits its ability to support causal inference where theory does not identify a clear direction. The potential downstream and upstream directions of interaction between some neighbourhood-level and individual risks make it difficult to determine the exact direction, especially for the person-level SES-related support pathway, of causation for these variables.

More specifically, poor person-level SES-related support is a direct reflection of poor individual SES. The interaction between neighbourhood-level SES and person-level SES involves both downstream and upstream impacts. A concentration of low SES residents may lead to neighbourhood deterioration or decay. In turn, deteriorated neighbourhoods comprise and affect local residents who are likely to be further socially excluded, which results in lower SES status. The relationship of neighbourhood health care and personal SES is also not completely clear. As suggested in the theories of political economy and landscapes of collective consumption [34,35], decisions on health service locations are made partly to favour groups or organizations that possess power, rather than reflecting the public “need”. It is therefore possible that concentrations of low SES residents locally may lead to an insufficient supply of health care in these areas. The need for confirmation of these complex interactions between person-level and neighbourhood-level risks makes it necessary to undertake future longitudinal or intervention-based studies to confirm causal directions, causal effects, and intervention outcomes.

Nevertheless, based on the likely nature of the effects of contributory factors on adverse birth outcomes, many causal directions are apparent, making it possible to draw general inferences regarding hypothesized causality. Hence, some conclusions can be made regarding the vertical (or top-down) pathways of neighbourhood-level SES related risks on adverse birth outcomes.

In particular, the results presented in this paper indicate that neighbourhood-level SES-related variables exert their impacts on adverse birth outcomes both directly and indirectly through the mediation of person-level risks. The direct associations indicate that neighbourhood risks, such as living environment and food access, are socially structured, and since a mother-to-be may have little control over these risks, the results suggest the necessity for direct community-oriented interventions. For example, by structuring service interventions to monitor healthy diets for expectant mothers in low SES areas may allow the associated risks of adverse birth outcomes to be reduced. Furthermore, providing primary prenatal care and social support in local communities may also help to reduce the risks associated with low neighbourhood SES on birth outcomes.

However, it is not practical to intervene directly on all identified community-level risks, as many of the contributory factors are systemic and require changes across many factors to have any visible impact. Social and financial supports within local communities involve the participation of multiple stakeholders beyond the health sector, and the level of involvement is difficult to determine in modern market-driven societies. Given the effects of prevailing globalization and social polarization processes on societies, the increasing social and economic gaps between the rich and the poor are not likely to be reversed in the short term. Hence, it is not practical to reduce neighbourhood socio-economic inequalities through the reduction of personal socio-economic inequalities. In fact, the increasing polarization of socio-economic classes has led to the division and segregation of residential areas and this has allowed socially isolated and economically deprived communities to develop.

The mediation analysis presented in this paper provides a theoretical basis, supported by empirical results, to identify and start to address neighbourhood factors alternatively through interventions on intermediate person-level risks. The results have demonstrated great complexities in the causal pathways through which neighbourhood-level SES-related determinants may affect adverse individual birth outcomes. However, it has been possible to identify numerous pathways and influences, both direct and indirect, that contribute to neighbourhood inequalities of PTB and LBW incidence. Given the generalization of person-level risk factors and consequently identified pathways in this research, these findings may be applied not only to the study areas but also to areas with similar neighbourhood conditions.

In addition to the direct effects, the results show that neighbourhood-level SES-related risks are largely mediated by material pathways, such as person-level SES-related supports. Additional material-related risks, such as the percentage of houses needing major repair, are also associated with PTB. However, a non-negligible portion of the indirect effects is apparent through psycho-social pathways, especially for LBW. The person-level behavioural and psycho-social pathways play more important roles in mediating neighbourhood SES-related risks for LBW than for PTB. Directly measured psycho-social risks, such as average level of emotional unhappiness, and indirect psycho-social-related risks, such as average person per room, person per bedroom and heavy drinking rates, are all associated with LBW. This suggests that procedures to improve neighbourhood social capital may have relatively more impact on reducing the incidence of LBW than on reducing PTB.

If the above-mentioned upward stream impact of person-level SES on neighbourhood-level health services, food supplies, and living conditions is accurate, a bottom-up approach of intervention may succeed. Such interventions could encourage local residents in socio-economically disadvantaged neighbourhoods to participate more actively in interactive decision-making designed to achieve the goal of improving local health care services and other health-related living conditions. Adaption of a bottom-up approach that involves aspects of local-level interventions with longer term health and general welfare policies to achieve systemic change is most likely to improve birth outcomes of local residents. In this approach, it is essential to identify the causal directions of the neighbourhood- and person-level associations in future research.

## Competing interests

The authors declare that they have no financial or non-financial competing interests.

## Authors' contributors

All of the authors contributed to the study design, writing and revision of the article. Dr. Meng designed and carried out the analysis. All authors read and approved the final manuscript.
